# Lupin (*Lupinus albus* L.) Seeds: Balancing the Good and the Bad and Addressing Future Challenges

**DOI:** 10.3390/molecules27238557

**Published:** 2022-12-05

**Authors:** Ana Pereira, Fernando Ramos, Ana Sanches Silva

**Affiliations:** 1 Faculty of Pharmacy, University of Coimbra, Azinhaga de Santa Comba, 3000-548 Coimbra, Portugal; 2 REQUIMTE/LAQV, Rua D. Manuel II, Apartado 55142, 4050-346 Porto, Portugal; 3 National Institute for Agrarian and Veterinary Research (INIAV), I.P., Rua dos Lagidos, Lugar da Madalena, Vairão, 4485-655 Vila do Conde, Portugal; 4 Center for Study in Animal Science (CECA), ICETA, University of Porto, 4050-346 Porto, Portugal; 5 Associate Laboratory for Animal and Veterinary Sciences (AL4AnimalS), 1300-477 Lisbon, Portugal

**Keywords:** lupine, white lupine, *Lupinus albus*, lupine seeds, nutritional value, protein, dietary fiber, bioactive compounds, anti-nutritional compounds, phomopsins, health benefits

## Abstract

*Lupinus albus* L. (lupine) is a legume whose grain/seed has gained increasing interest. Its recognized nutritional properties, namely a high content of protein, dietary fiber and its low fat content, make lupine a suitable alternative not only for animal protein, but also as a substitute for more processed and less balanced flours from a nutritional point of view, used in the preparation of bread, cakes and cookies, among others. In addition, its nutritional and bioactive compounds have potential benefits for human health in the prevention and treatment of some diseases. However, the existence of some anti-nutritional compounds and contaminants reveal some concern, requiring effective methods for their detection and eventual removal. This review intends to address the potential of lupine (*L. albus*) in food and human health and to balance the pros and cons. Nutritional and anti-nutritional components of *L. albus* seeds and possible contaminants of lupine seeds are examined. The potential health benefits of lupine (seeds), including energy metabolism, cardiovascular diseases, hypertension, glucose and insulin metabolism, bower function and anticonvulsant action, are discussed based on scientific evidence (both clinical trials and studies performed with animal models).

## 1. Introduction

In recent times, there has been a growing interest in plant-derived nutrients and bioactive compounds, not only due to economic and environmental factors but also due to the need to develop new, safe and healthy foods that can respond to the growing awareness and interest in healthy eating habits [1].

In this sense, the interest for sweet lupine seeds is continuously growing, stimulated by its flexibility in food preparation as well as by the increasing knowledge of the health benefits they provide [2]. Among the species of the genus Lupinus, the white lupinus, *Lupinus albus* L., has notable potential. The production of this genus of legume is increasingly recurrent, not only because of the possibility of cultivation in poor soils and under adverse conditions, but also due to its use in animal nutrition (feed or protein supplementation for ruminants) and by humans, because seeds (the most commonly used part of lupin) have high protein and oil content. On the other hand, soil fertility can be improved by supplementing poor soils with nitrogen compounds [3,4].

One of the reasons for the increased interest in this legume is the numerous studies that show that it provides positive health benefits, particularly in the area of dyslipidemia, hyperglycemia and hypertension prevention [2]. These benefits are related to their content in bioactive compounds, with antioxidant and anti-inflammatory properties [5].

**Figure 1:**
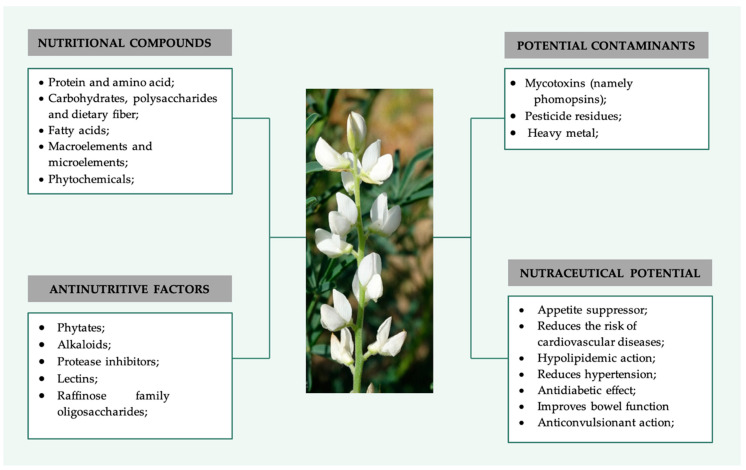
Summary of the main components of *L. albus* including nutrients, bioactive and antinutritive factors and potential contaminants, as well as main nutraceutical potential of this legume. (Photo of the species from the Botanical Garden UTAD, Digital Flora of Portugal).

Lupine was chosen for this review as it (i) is a good break crop because it has the ability to fix nitrogen, (ii) it adapts well to different environmental conditions, which is an important feature due to climatic changes, and due to (iii) its high content of protein and bioactive compounds with valuable biological properties. This appraisal review intends to compile the most valuable information regarding *Lupinus albus* seeds, namely its nutritional and anti-nutritional components. Moreover, it also addresses the possible contaminants of lupine seeds, focusing on mycotoxins. Finally, the potential health benefits of lupine seeds are discussed based on the scientific evidence of clinical trials and studies carried out with animal models (Figure 1). The by-products of *L. albus* are not addressed in this review.

## 2. Lupin (*Lupinus albus* L.)

### 2.1. General Description

White lupin, the common name for *Lupinus albus* L., is a species of the genus Lupinus, tribe Genisteae, family Leguminosae or Fabaceae. This type of Lupin is a non-native cultivated, annual legume that can reach a height of approximately 120 cm. It also has a strong stem, which in combination with the secondary roots, can penetrate the soil to a depth of 1.5 m [6]. Leaves are alternate (which means that each node has only one leaf) and with five to nine leaflets, nearly smooth above and hairy beneath. Individual plants produce several orders of inflorescences and branches, resulting in clusters of long, oblong pods, each cluster having three to seven pods, and each pod containing three to seven seeds [7].

Like most of the members of their family, white lupins can fix the nitrogen provided by the atmosphere into ammonia. This allows the fertilization of the soil for other plants and allows lupins to be tolerant to infertile, acidic and sandy soils and able to improve the quality of poor soils [8].

White lupine flowers in May–June. The flowers are white to violet, with the upper lip being entirely three-toothed and the lower lip being entirely or slightly three-toothed. The seeds are large, cream in color with a circular flattened shape, and with a 1000-seed-weight of 350–400 g [8].

Furthermore, white lupin is primarily a cross-pollinated crop, but self-pollination of 50–85% has been reported [7]. Due to this biological floral characteristic, sweet Lupine is selected for the production of pure lines [9]. Bitter and sweet forms of white lupin exist, but the bitter forms have a high alkaloid content, and for this reason, the sweet form is preferable to be cultivated and consumed [6]. However, although sweet lupine is more advantageous for the food industry, bitter lupine can fix more nitrogen, providing an alternate use of inorganic N fertilizers. In a study conducted by Staples et al., it was observed that white lupine lines with higher seed alkaloid concentrations (bitter lines) had higher root nodulation for symbiotic N fixation. Furthermore, in this same study, by characterizing the concentration of alkaloids in leaves, pod shells and seeds of white lupine (*L. albus*), it was possible to perceive that some strains had different alkaloids in seeds, leaves and pod shells. This indicates that it may be possible to develop lupine strains with sweet or bitter seeds, each individually combined with different alkaloid contents in the leaves or pods. The significance of these results is that it may be possible to develop strains of lupine that have sweet seeds for use as food or feed, while the remaining plant tissue contains high levels of alkaloids to make it suitable for helping to control insect pests or destructive microorganisms [10].

### 2.2. Use of the Plant over Times

The white lupin is a species mainly distributed around the Mediterranean and along the Nile valley. In these areas, it has been traditionally cultivated for several thousand years [9].

Lupins can be used for many purposes. These include food, pasture improvement, ornamentation, erosion control and soil stabilization. Furthermore, the bitter species can be used to control some pests due to its alkaloid content [11].

Historically, Egyptians, Romans and Greeks have used lupine as a soil fertilizer since this plant has the ability to grow in poor, acidic, sandy soils and in adverse weather conditions. In pre-Columbian times (before the arrival of Europeans in America), lupine was used as a food source for these populations; it was soaked and then cooked to remove the bitter taste derived from the presence of alkaloids (quinolizidine alkaloids) in the lupine [12].

In the modern age (15th to 18th centuries), *L. albus* was introduced in Germany. The results of introducing this plant as a food source were poorer than expected, but the cultivation of lupines in the sandy coastal plains of the Baltic prospered. It was used predominantly as animal feed and as soil enrichment due to its nitrogen fixing properties. However, until the First World War, the emergence of toxicity associated with lupine in sheep led to a decrease in interest in its cultivation [12].

During and after the first war period in Germany, due to the food shortage, the attention given to lupine was redoubled as it constitutes an alternative source of protein. With increasing interest in lupine, research on reducing the alkaloid content of lupine caught the attention of some scientists at the Kaiser Wilhelm Research Institute, who managed to reduce the alkaloid content from the traditional 1–3% to less than 0.02% [12].

In addition to the important uses of lupine both in food and in agriculture, lupine had a relevant role in the traditional medicine of several civilizations. For example, it was used as one of the many traditional remedies of the civilizations of Jordan and Greece for the treatment of diabetes. Furthermore, its use was also reported for conditions such as abscesses, parasites, heart disease and rheumatism [13].

A role in epilepsy has also been proposed [13]. In the Middle Ages, a decoction was used to treat seizures, a condition that was associated with devil possession and wolves. Several studies have been carried out to understand the evidence of this use and the truth underneath. Studies were carried out in rats, where three extracts rich in lupine alkaloids (sparteine, cytisine, lupinine) were administered. It was noticed that these three alkaloids produced a non-specific depressant response of the central nervous system (CNS) in rats, especially at higher doses [14]. In Section 6, the most relevant studies addressing the role of lupine alkaloids in CNS is addressed.

The interest in the production of *Lupinus albus* has been growing recently due to its potential as a source of protein, its potential pharmaceutical purposes, the fact that it can be grown in a wide range of climatic conditions and due to the high content of alkaloids that, despite being toxic, when well isolated and formulated, can have beneficial effects on health, and can work as a natural pesticide for the plant itself [15]. Furthermore, its adaptation to poor soils makes lupine an economically viable plant [16]. Lupine is commonly consumed as a snack in the Middle East and is being used as a high-protein soy substitute in other parts of the world. For example, in Europe, lupine seeds have been used for many years to replace cereal grains in flours and pasta or are sometimes used as complete or partial substitutes for soybeans in the production of liquid or powdered vegetable drinks and tofu [17].

However, although it is well known, widely cultivated and consumed by people living in Europe, its cultivation lags behind other legumes [17]. However, from the year 2000 (88.091 t/y) to the year 2020 (457.963 t/y), a high growth in lupinus production in Europe was visible, which demonstrates the growing interest in lupines as food and feed. According to FAOSTAT, in 2020, the main lupine-producing countries in Europe were Poland (261.500 t/y), Germany (34.100 t/y), France (12.820 t/y) and Spain (2470.00 t/y) [18].

## 3. Nutritional Compounds of Lupin Seeds

Among legume seeds, lupine seeds are some of the most appreciated since they are a good source of nutrients, mainly proteins, but also lipids, dietary fiber, minerals and vitamins [19,20].

The most commonly consumed part of the lupin plant is the seed. After the alkaloids are removed from seeds by debittering or by breeding to produce sweet varieties, lupin seed is highly valuable as a human food and animal feed, with high protein and dietary fiber and low levels of antinutritional factors such as phytates, protease inhibitors and lectins [21].

According to the National Institute of Health Dr. Ricardo Jorge (INSA) and the Portuguese food composition database (PortFIR), cooked and unsalted lupine is mostly made up of water (68.6 g/100 g), protein (16 g/100 g), carbohydrates (7.2 g/100 g), fiber (4.8 g/100 g) and lipids (2.4 g/100 g) [22]. According to the United States Department of Agriculture (USDA, Washington, DC, USA) the values are as follows: water (71.1 g/100 g), protein (15.6 g/100 g), carbohydrates (9.88 g/100 g), fiber (2.8 g/100 g), lipids (2.92 g/100 g) [23].

### 3.1. Protein and Amino Acids

In general, grain legumes are good sources of vegetable protein, among which *Lupinus albus* is known to have seeds with high protein content, like soybean [15,24]. Based on this fact, *L. albus* seeds have been employed as a protein source for animal and human nutrition in various parts of the world [17,25].

Lupine proteins have raised a lot of interest due to their functional and nutritional impact on the products in which they are included. Some of these functional properties include the protein solubility, swelling, foaming and gelling propensity.

On the other hand, several studies (some cited in Section 6) have revealed promising applications of lupine proteins in the treatment of health disorders, namely due to their influence on lipid and glucose metabolism, as well as blood pressure levels. Another interesting aspect of lupine protein functionality is its possible effects on inflammatory processes and changes in the gut microbiome, which has a significant influence on several physiological parameters, including metabolism, nutrient absorption and immune function. Protein content of lupins is approximately 34–44% on a dry weight basis (dw) which is almost similar to soybean (12.5 g/100 g) and is significantly higher than other legumes such as lentils (9.1 g/100 g), pea (6.2 g/100 g), chickpea (8.4 g/100 g) and faba bean (6.7 g/100 g) [1,2,26,27,28,29].

Apart from quantity, the quality of protein is mainly based on its amino acid composition and protein digestibility. Thus, it is important to consider not only the total amount of protein but also the amino acid composition. As a member of legume family, lupin bean protein is deficient in sulfur-containing amino acids, methionine and cystine. They are also relatively low in valine and tryptophan, and high in lysine. Lupine protein is high in glutamic and aspartic acid and arginine [12,17,27].

In lupin seeds, the two most important lupin proteins are the albumins and globulins, in a relative ratio of about 1:9 [30]. The globulins correspond to about 90% of the total protein content, with albumins making up the remainder [1,31]. Based on the electrophoretic mobility (the α-, β- and δ-conglutins are acidic while γ-conglutins are basic proteins) of lupin seeds [32]. Proteins may be separated by ultracentrifugation and chromatography into two main components: the two major storage proteins, α-conglutin (11S legumin-like lupin globulin) and β-conglutin (7S vicilin-like lupin globulin), and two minor groups, γ-conglutin (7S globulin family) and δ-conglutin (2S sulfur-rich albumin family) [1,33]. β-conglutin is the most abundant lupin globulin, representing nearly 44–45% of white lupin seed storage proteins, followed by α-conglutin with 35–37%. The content of γ-conglutin in mature white lupin seeds accounts for about 4–5% of the total amount of proteins, while the least present is δ-conglutin, representing only around 3–4% of total conglutins in white lupin [1,27].

### 3.2. Carbohydrates, Polysaccharides and Dietary Fiber

In contrast to other vegetables, lupine seeds are virtually starch-free, which makes lupine carbs nutritionally interesting [30]. This starch, which is present in low amounts, can be classified as resistant starch, meaning it is slowly digested and thus releases glucose gradually into the blood [34]. Therefore, most of the carbohydrates that prevail in mature seeds are oligosaccharides and non-starchy polysaccharides, particularly from the cell wall structure. The oligosaccharides found in the cotyledons are sucrose and nondigestible galactosides of the raffinose family [34].

The polysaccharides are present as major components of the cell wall surrounding the cytosol. Due to the polysaccharide composition, lupins are rich sources of fiber. Dietary fiber represents 40% of the kernel weight of white lupine, which is a higher level than in most other vegetables, and it has the lowest glycemic index of any commonly consumed grain [17,35]. The seed cover of *L. albus* after the debittering process contains 89% of insoluble dietary fiber. The main component of the insoluble dietary fiber is cellulose (79%). Others include hemicelluloses and lignin, remaining at levels of 14% and 7%, respectively [19,36].

Due to these characteristics, lupine carbohydrates may provide useful material to produce special high-fiber-content food for humans. This fiber incorporated into food products can benefit intestinal functions and fecal parameters by decreasing the risk of colon cancer and promoting a healthy digestive system, and has the capacity to decrease cholesterol levels [37,38].

### 3.3. Fat Content and Fatty Acids

Lupine seeds have a significantly low fat content, with white lupine having a fat content of 2–3 g/100 g [38,39].

Although lupine is not described as an oilseed, it has a considerable amount of oil in its seeds. It contains approximately 5–20% of crude oil in the whole seed [19]. The oil extracted from *L. albus* seed consists of various types of fatty acids. The fatty acids of the oil from the raw seed are mainly composed of unsaturated fatty acids but also a small percentage of saturated fatty acids [11], which means *L. albus* can be a potential source of useful vegetable fat. 

Among the unsaturated fatty acids, oleic (ω-9, 18:1) and linoleic (ω-6.18:2) are the predominant fatty acids, followed by alpha linolenic acid (ω-3, 18:3). Particularly, *L. albus* contains a high level of linolenic acid [27,34,40,41].

A low amount of SFA (saturated fatty acids) and high amount of PUFA (polyunsaturated fatty acids) level in the diet is considered beneficial to health; especially, the ratio between ω-6 and ω-3 fatty acids is an important determinant in the prevention of several metabolic diseases, including coronary heart diseases. The ratio of PUFAs to SFAs in lupine seeds is in the range of 1.3 to 2.9:1. Higher rates are important, since it has been strongly recommended that a reduction in SFAs in favor of an increase in PUFAs in the diet should be achieved, to assist coronary heart disease prevention [27,34,41].

### 3.4. Other Macro and Micronutrients

Although white lupine is an interesting legume, current information and research regarding the presence of minerals and vitamins are scarce [27].

White lupine seeds are a rich source of macro and micronutrients (Table 1); their total content is 30–40 mg/kg [15,42]. Typical mineral contents reported in white lupin seeds are calcium, from 2.1 to 4.7 g/kg; phosphorus, from 4.3 to 7.2 g/kg; magnesium, from 1.2 to 2.2 g/kg; potassium, from 8.6 to 11.1 g/kg; and sodium, from 0.1 to 0.2 g/kg [43]. In terms of manganese, a high content (896 mg/kg) was detected in *L. albus* [16,32,38].

Lupin seeds also contain vitamins (Table 1) such as thiamine, niacin, riboflavin and tocopherols, as well as carotenoids [32]. According to some authors, the consumption of 100 g of lupin seeds is capable of satisfying approximately 50% of thiamin (B1), 30% of niacin (B3) and 20% of riboflavin (B2) requirements for a diet of 2000 kcal/day [4]. In terms of vitamin E, γ-tocopherol is the main isomer of vitamin E in lupins, and compared to other species of Lupin, Lupin albus is one of those that have the highest content [24]. The presence of vitamin C was only detected in *L. albus* and *L. luteus* [28,29]. Relative to carotenoids, the presence of zeaxanthin, lutein and β-carotene in lupins has been reported [27,28,29,38].

### 3.5. Phytochemicals

Phytochemicals are a group of plant-derived compounds, and some of them play an important role in disease prevention through the consumption of diets rich in fruits, vegetables and legumes, among others. Based on their biosynthetic origin, these compounds can be classified into several groups, for example, polyphenols, phytoestrogens, terpenoids, carotenoids, limonoids, phytosterols and phytohemagglutinins [5].

Lupine seeds have significant amounts of phytochemicals, importantly polyphenols, phytosterols and squalene (triterpene) [44]. These compounds, together with fiber, may protect against chronic diseases including cancer and cardiovascular disease [32].

#### 3.5.1. Polyphenols

Most of the phenolic compounds identified in lupine belong to the subclasses of flavones, phenolic acids and isoflavones. Phenolic compounds can be found in all parts of lupine, i.e., stem, leaves, roots and seeds, and their concentrations vary depending on the part of the plant. The total content of phenolics varied from 212 to 318 mg per 100 g dw expressed as gallic acid equivalents in the seeds of *L. albus* [5].

**Figure 2:**
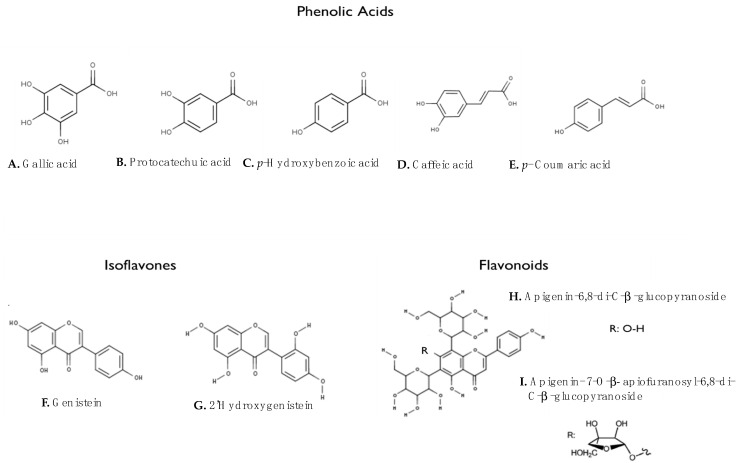
Structures of the main polyphenols in lupin seeds.

According to Arnoldi et al. [2], the phenolic acids are gallic acid (3.5 mg/kg in white lupine) (Figure 2A), protocatechuic acid (13.8 mg/kg in white lupine) (Figure 2B), p-hydroxybenzoic acid (25.3 mg/kg in white lupine, white lupine) (Figure 2C), caffeic acid (0.3 mg/kg in white lupine) (Figure 2D) and p-coumaric acid E (0.1 mg/kg in white lupine) (Figure 2E). Isoflavones are represented by genistein (Figure 2F) and 2′-hydroxygenistein (Figure 2G), at concentrations of 3–5 mg/kg and 1–5 mg/kg, respectively. The main flavonoids are two C-glycosides of apigenin, namely apigenin-6,8-di-C-β-glucopyranoside (131 mg/kg in white lupine) (Figure 2H) and apigenin 7-O-β-apiofuranosyl-6,8-di-C-β-glucopyranoside (258 mg/kg in white lupine) (Figure 2I), which are the most abundant polyphenols in this seed. Compound I of Figure 2 is a rare type of glycoside, which was mainly detected in Lupinus [2].

#### 3.5.2. Phytosterols

Phytosterols are the analogous compounds of cholesterol in animals, which are mainly present in nuts, oils, cereals and vegetable grains. This similarity between phytosterols and cholesterol allows them to relocate low-density lipoprotein cholesterol in the human intestine, which is why these compounds are associated with cholesterol reduction as well as anticancer and anti-inflammatory activities [5,32].

There is limited information available on phytosterol contents in different lupine species; however, the phytosterols that are present in greater amounts are β-Sitosterol and stigmasterol [5]. Some studies reported that phytostoral production is conditioned by abiotic and biotic factors. In a study conducted by Hamama et al., it was noticed that the amount of β-sitosterol produced by *L. albus* varied according to temperature, concluding that lower temperatures during lupine growth stimulated β-sitosterol synthesis and inhibited the synthesis of stigmasterol. On the other hand, high growing temperatures reversed this trend. Therefore, a higher β-sitosterol/stigmasterol ratio should maintain membrane fluidity under low temperatures, decreasing water loss under cooling conditions. Furthermore, metabolic alterations in β-sitosterol and stigmasterol levels have also been associated with fungal or bacterial infections and have been related to the induction of signaling pathways that lead to the synthesis of antimicrobial molecules and changes in membrane permeability [45,46].

#### 3.5.3. Tocopherols

Tocopherols, commonly called vitamin E, are some of the most effective lipid-phase natural antioxidants, being able to inhibit lipid peroxidation by acting as peroxyl radical scavengers that stop the chain reactions in membranes and lipoprotein particles [5,47].

Most tocopherols present in lupine are α-tocopherol, γ-tocopherol and δ-tocopherol [5,48].

These tocopherols are temperature-sensitive, so cooking the seed of *L. albus* causes significant loss of tocopherols, resulting in concentrations of δ-tocopherol as low as 0.02 mg per 100 g fresh weight [49]. Tocopherols present in *L. albus* seeds could be extracted and used to fortify foods with natural antioxidants or be applied in cosmetics or pharmaceuticals.

#### 3.5.4. Other Phytochemical Compounds

Other phytochemical compounds found in lupine include triterpenes such as squalene, triterpenic acids (ursolic acid and oleanolic acid) and triterpene alcohols such as lupeol and β-amyrin. Triterpenes have anti-inflammatory, hepato-protective, antitumor, antiviral, anti-HIV, antimicrobial, anti-fungal, antidiabetic, gastroprotective and antihyperlipidemic pharmacological potential, but there is still little information regarding these compounds in *L. albus* seeds [5].

## 4. Antinutritive Factors

One of the main problems arising from the use of plants as sources of nutrients in the diet is the presence of some compounds derived from their secondary metabolism. Anti-nutritive compounds can have detrimental effects on human growth and performance by impairing the intake, absorption or utilization of other foods or causing discomfort and stress in humans [32,50,51].

Unlike other legumes (peas, soybeans and beans), white lupine seeds are characterized by a low or very low content of anti-nutritive substances such as phytic acid, alkaloids, oligosaccharides, trypsin inhibitors, lectins and saponins. The removal of these substances can be achieved either through the selection of genotypes with low levels of these compounds but also through different techniques such as germinating, cooking, soaking, fermentation or selective extraction [19,24,51,52].

### 4.1. Phytates

Phytic acid, or phytate, is considered an anti-nutritive compound because it is related to a reduction in the bioavailability/absorption of some minerals such as Zn, Ca, Fe, K and Mg through the chelation of cations for non-absorbable phytinians [51]. Zn is the most affected, as demonstrated by several studies both in animals and humans. It has been shown that calcium potentiates the negative effect of phytate on Zn absorption [16].

The phytic acid content of the lupine seeds ranges from 0–4 to 1.2 g/100 g dw, with little variation within each species. White lupine is one of the species with the lowest phytic acid content, with this value being less than 0.7 g/100 g dw. Its content can be further reduced through fermentation or seed extrusion [16,51].

### 4.2. Alkaloids

The main alkaloids present in lupine belong, in general, to the quinolizidine family. Quinolizidine alkaloids (QAs) are a family of about 100 bitter components—secondary metabolites of a bicyclic, tricyclic and tetracyclic structure. The content of these alkaloids varies according to the variety of the species, i.e., the bitter or wild species and the sweet ones. In bitter cultivars, the alkaloid content ranges between 0.5% and 6%, and in sweet cultivars, it is less than 0.02%.

**Figure 3:**
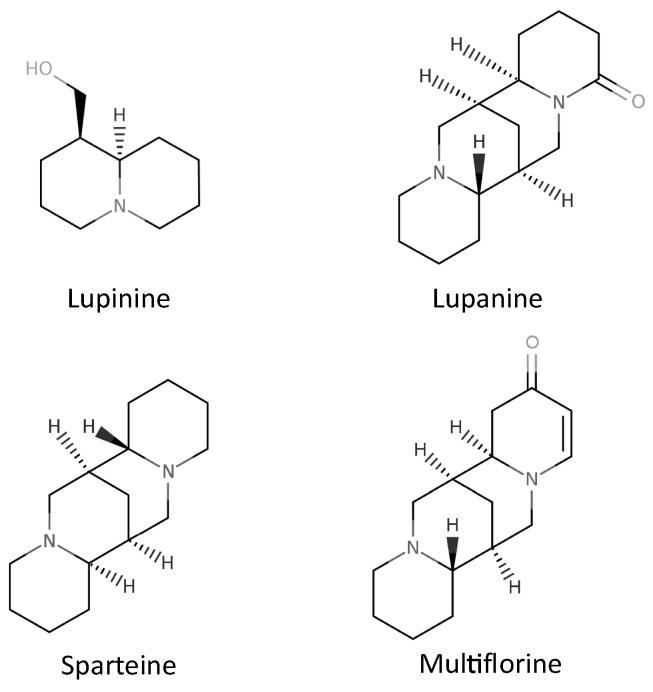
Main alkaloids in seeds of *Lupinus albus*.

In the seeds of *L. albus*, the major alkaloids present are lupanin, hydroxyaphylline, albine and multiflorine (Figure 3). Sparteine, albine and anagraine are also found [4].

For the plant itself, the alkaloids work as a means of protection against herbivorous animals, as they are bitter and toxic to these animals. Furthermore, during plant development, the alkaloid concentration in different plant parts (leaves, roots, stems) changes, reaching the highest value in the flowering stage [51].

On the other hand, for humans and animals, the QAs have received much attention, because they have a strong bitter taste and may be toxic in high doses [34]. QAs display similar agonistic activities as the alkaloid nicotine, i.e., they also affect Na^+^ and K^+^ channels, inducing gastrointestinal, nervous and respiratory symptoms [17,25]. General toxic symptoms caused by quinolizidine alkaloids may include malaise, nausea, respiratory arrest, visual disturbances, ataxia, liver damage, progressive weakness and coma [4,36].

To avoid serious health problems with the consumption of these alkaloids in lupine, several countries have established maximum limits for the presence of these compounds in products containing lupine. For example, France, Great Britain, Australia and New Zealand established a level up to 200 mg/kg seeds [3,53]. However, at a general European level, according to Annex of Commission Regulation (EC) No. 1881/2006, where maximum levels (MLs) for the same contaminants in different foods are established, currently, no MLs are set for QAs in food [54]. These limits are required by Article 2 of Council Regulation (EEC) No. 315/93 [55], which states that foods containing a contaminant in amounts unacceptable to public health must not be placed on the market, that contaminant levels must be maintained as low as possible, and that if necessary, the European Commission may set maximum levels for specific contaminants [54,56].

Thus, for lupine to be consumed safely, it is necessary to remove these alkaloids, which can be achieved through leaching (successive washing of the seeds with water), which is the most common method in Portugal. However, leaching, in addition to being inefficient, leads to the loss of nutritional value of these seeds, namely the loss of proteins. The second method, the most prevalent in most European countries, is the cultivation of “sweet” lupins (such as White Lupin), which produce alkaloids in insignificant amounts and therefore, their seeds do not need to be subjected to leaching [3].

Although the alkaloids found represent a problem with regard to the food industry, they can also be seen as a potential source of valuable active molecules for the pharmaceutical industry, having already been described as having antimutagenic, antibacterial, antifungal, anticancer and anti-inflammatory properties [57]. In addition, they can also be used as chiral synthons in the synthesis of more complex molecules, as already described for lupanine [3].

It was also found that lupanine potentiates the release of insulin by glucose, becoming a tool in the treatment of type II diabetes [58], and that sparteine (alkaloid present in small amounts in lupine, which can be easily obtained from lupanine) has the ability to be anticonvulsant [59].

### 4.3. Protease Inhibitors

Protease inhibitors are proteins that bind strongly to digestive enzymes, such as trypsin, impairing their activity by reducing their digestibility, which in turn will lead to malnutrition.

On the other hand, in plants, these inhibitors constitute a form of protection against damage inflicted by animals, insects and microorganisms [30].

The content of these inhibitors present in lupine seeds depends on each species, and in the case of white lupine, they are mostly undetectable, i.e., less than 0.1 mg/g. Although present in small amounts, protease inhibitors may be destroyed by heat treatments [33].

### 4.4. Lectins

Lectins are a class of glycoproteins capable of agglutinating red blood cells and preventing the absorption of nutrients through their ability to bind to wall epithelial cells, causing deleterious nutritional effects. White lupine and other lupine species contain small amounts of lectins compared to other vegetables, such as soybeans [30,33].

### 4.5. Raffinose Family Oligosaccharides

Like other legumes, lupine contains raffinose family oligosaccharides, although, compared to other legumes, they are in lower amounts. α-galacto oligosaccharides raffinose family oligosaccharides (raffinose, stachyose, verbascose, etc.) are characterized by the presence of (one to six) links between galactose residues [17,30]. The enzyme α-galactosidase, which is necessary for the hydrolysis of α-1,6 bonds, is unavailable in the small intestine of animals and humans. As a result, these compounds pass into the large intestine where they undergo a process of fermentation and gas production. A consequence of these processes is the formation of CO_2_, methane and H_2_, which cause the typical symptoms of stomach ache and flatulence, from which people can suffer after consuming a high amount of legume seeds [51].

Despite being considered anti-nutritional compounds in excess, the raffinose family of oligosaccharides has been increasingly used to produce probiotics and prebiotics. Some studies also indicate potential value for the immune system, i.e., antioxidative activity and antitumor activity, as well as lowering the cholesterol level [51].

## 5. Potential Contaminants of Lupine Seeds

### 5.1. Mycotoxins

Like practically all plants, white lupine is also subject to contaminating factors from the external environment, such as common slugs that attack leaves and fungal pathogens such as *Ascochyta* sp., Fusarium avenaceum, Fusarium oxysporum, Pleiochaeta setosa, Septoria glycines and *Colletotrichum* sp., which are easy to identify and eliminate using fungicides, and do not have a significantly harmful effect on the seed consumer [7].

On the other hand, one of the worrying contaminants of lupine that led the European commission to ask EFSA to assess the risks to human and animal health related to these substances are Phomopsins (PHOs). PHOs are a group of linear hexapeptides from a family of mycotoxins produced by the fungus *Diaporthe toxica*, with an antimicrotubule effect, which is mainly pronounced in the liver and kidneys. They are able to interrupt mitosis and reduce the activity of cells and organisms, making their host susceptible to secondary infections. Lupine is one of the biggest hosts for this fungus, capable of infecting most parts of the plant [60,61,62].

Once contaminated, the seeds become toxic and the amount of PHOs can increase under certain storage conditions. As lupine products have become increasingly involved in human nutrition, whole lupine seed and flour may be a source of human exposure to phomopsins, which have been shown to be stable with processing, including cooking, and are therefore a worrisome contaminant [61].

#### 5.1.1. Formation of Phomopsins in Lupine

The lupine plant becomes infected by Diaporthe toxica during the growing season, with the stem being the most infected part. Some studies indicate that the seed infection originates in the pod, with the mycelium penetrating the inner wall of the pod and infecting the seed coat, or by systemic growth of the fungus from pod stalk lesions. When the seed infection is asymptomatic (most often it is), the fungus only remains in the seed coat and the seed is able to grow normally [61].

On the other hand, in a symptomatic infection, an infected seed is characteristically discolored, ranging from pale orange-yellow to dark reddish-brown, with white mycelial growth sometimes being evident on the darkly discolored seeds. Discolored seed is only found in pods with visible lesions of infection, it is smaller and lighter than normal, the kernel is also discolored, the fungus colonizes all tissues of the seed and the seed is not able to germinate [61].

In cases where phomopsins are produced on discolored seeds, there is a directly proportional relationship between the content of phomopsins in the seed and its blacker color, i.e., the darker the seeds, the higher their phomopsins content. Furthermore, the highest concentration of phomopsins is in the lupine seed husk, but this does not mean that the kernel is not toxic in some cases [61].

The ideal conductions of temperature and humidity for fungal growth are not known; however, in the laboratory, it is estimated that 25 °C is the optimum temperature for toxin production in liquid culture [61].

The amount of phomopsins in food can be analyzed by analytical methods such as enzyme-linked immunosorbent assay (ELISA), high-performance liquid chromatography (HPLC) and fast atom bombardment of solids (FAB) mass spectrometry [61].

In the ELISA, the quantification of phomopsins in sample extracts is detected by the competitive inhibition of the reaction between a phomopsin–peroxidase enzyme conjugate and solid-phase anti-phomopsin antibodies coating the wells of the microtiter plate. The concentration of free phomopsins present in the sample is directly proportional to the amount of inhibition of the phomopsin-peroxidase conjugate. The degree of inhibition, compared to uninhibited controls, is visualized by the intensity of color development after the addition of 3,3′,5,5′-tetramethylbenzidine (TMB) substrate [61,63].

In HPLC, the basic principle of chromatography is applied, the existence of a mobile phase, which in this case is liquid, and a stationary phase. However, despite theoretically being a simple method, it was found that impurities with similar retention times of phomopsins were absorbed and consequently interfere with the assay, reducing both the specificity and sensitivity of the method. For this, it is necessary to wash the samples before carrying out the analytical process, which consumes a lot of time [61,63].

FAB mass spectrometry has been used to confirm the identity of phomopsins to determine the amino acid constituents and amino acid sequence of these mycotoxins [61].

The occurrence of phomopsins is not covered by European Regulation (EC) No. 1881/2006, where MLs are established for certain contaminants in food [54] (Commission of the European Communities, 2006). However, some MLs for various mycotoxins were set for a number of food commodities [61].

The only country that has MLs for the presence of phomopsins is Australia, where the limit for phomopsins in lupine seeds and products is 5 μg/kg, set out by the Australian and New Zealand Food Authority in the Food Standards Code—Schedule 19—Maximum levels of contaminants and natural toxicants [64,65].

In addition to Australia, the presence of phomopsins caught the attention of the U.K., which led the U.K. Committee on Toxicity in Foods, Consumer Products and the Environment to conclude that a limit should be set for phomopsins in final lupin products. The U.K. Advisory Committee on Novel Foods and Processes subsequently recommended a limit of 5 μg phomopsins/kg in the final lupine product [61,62,66].

#### 5.1.2. EFSA Recommendations

Data regarding the presence of phomopsins in lupin-based food and feed are limited, which prevents conclusions regarding the assessment of dietary intake of phomopsins. Thus, there are no data describing the gastrointestinal absorption, metabolism, tissue distribution and excretion of phomopsins in animals and humans, as well as the mechanism of action by which phomopsins act, which is thought to be through modulation of microtubular function due to high-affinity binding of phomopsins to tubulin isotypes [61].

Although the scarcity of data on dose–response relationships for toxicities associated with phomopsins does not allow an assessment of human and animal risks, and according to the severity of effects on liver and kidney among all animal species tested, EFSA suggests that human and livestock exposures should be kept as low as possible [61].

In addition, EFSA also suggests an increased effort to develop analytical methods for the detection of the major toxic phomopsins and also to conduct studies that, through analytical methodology, generate sufficient additional information about human exposure and mechanisms of action [61].

### 5.2. Other Contaminants

The contamination of agricultural soils and the presence of chemical residues in agricultural products have raised concern, and consequently, there has been greater awareness and public concern about the quality of food and soil [67].

One of the worrying examples is the presence of cadmium (Cd) in the growing soil of lupins. Cd is a dangerous heavy metal easily taken up by plants, and usually causes injury even at low levels [68]. Excess Cd in the plant can affect many plant processes, including transport across membranes and photosynthesis, and it is associated with disturbances in the uptake and distribution of plant nutrients [69].

A possible source of contamination of soils and plants with Cd is the use of phosphate pesticides made from phosphate rock that contains Cd impurities. Regular use of phosphate fertilizers (mainly superphosphate containing 9.1% P and 11% S) for agricultural production, combined with the physical (sandy) and chemical (low soil pH, low cation exchange capacity) properties of the soil, constitute a concern for the high presence of this heavy metal in lupine grains [67].

To ensure the safety of lupine consumers, the Codex Alimentarius limited the presence of Cd in pulses to a value of less than 0.1 mg/kg [70,71].

## 6. Nutraceutical Potential for Human Health

Over the years, it has been discovered that lupine and lupine-enriched foods can influence health and the prevention of certain diseases (Table 2). White lupine has shown great potential in situations such as causing feeling of satiety (appetite suppression) and affecting the energy balance, favorably affecting glycaemia, improving the levels of blood lipids, having a positive effect on hypertension and improving defecation [38].

### 6.1. Effect on Satiety (Appetite Suppression) and on the Energy Intake

There is clinical evidence that foods containing lupine or lupine-derived compounds may reduce appetite after eating, which may lead to a decrease in food intake and consequently may influence the maintenance of a healthy body weight. For example, in a study conducted on 88 healthy adult volunteers over 16 weeks, it was observed that a breakfast with the same energy as wheat bread containing 40% lupine flour gave higher levels of self-reported satiety and lower energy intake at lunch than compared to bread made with wheat flour alone [83].

Another study with 20 healthy adults revealed that they also found a greater perception of self-reported satiety after eating lupine bread than wheat-only bread [73]. In another study conducted by Archer et al. [74], the incorporation of lupine seed fiber into processed foods resulted in feelings of fullness for up to 4.5 h after ingestion and in approximately 15% lower energy intake [28,29,38,74].

However, despite the effect of satiety being visible and evidence that a high-protein diet compared to a high-sugar diet or a high-fiber diet compared to a low-fat diet fiber is more filling and has an effect on decreasing food intake, the satiety effect of lupine foods has not translated into actual effects on weight loss in clinical studies. Thus, it is necessary to better understand and explore how to use lupine for this effect and which mechanism or compound is responsible for this satiety effect [28,29,38].

### 6.2. Role as Cardiovascular Disease Prevention

Cardiovascular disease (CVD) is the leading cause of death in developed countries. The influence of lifestyle and genetic predisposition are the biggest risks for the appearance of CVD. In economic terms, these diseases represent a high expense in the area of health care. Thus, preventive measures such as healthy eating are necessary [33].

The effect of the lipid profile of lupine-derived proteins remains somewhat uncertain, although there are several studies that point to a role in reducing the occurrence of atherosclerotic lesions and cholesterol levels. In a study in animal models (mice), comparing the proteins of white lupin administered to mice with atherosclerosis to the control group fed with casein, the proteins of white lupine reduced the occurrence of atherosclerotic lesions [75]. A randomized study also showed that the addition of 17 to 30 g/d of fiber derived from lupine to the diet reduces total and LDL cholesterol by about 5% [76,84,85].

Part of this activity can be explained by an increase in the expression and activity of SREBP-2, a transcription factor that upregulates the LDL receptors (LDL-R) in hepatocytes. Furthermore, Bettzieche et al. and Parolini et al. reported that the expression of CYP7A1, a rate-limiting enzyme that converts cholesterol to bile acids, was also regulated by the lupine protein diet, suggesting that the cholesterol-lowering effect was also due to an increased excretion of bile salts in feces [33,77,78].

### 6.3. Role in Arterial Hypertension

Arterial hypertension is a known risk factor for cardiovascular diseases, and there are several studies that demonstrate a relationship between blood pressure values and several nutrients, among which proteins and fibers stand out [83].

An increase in protein intake, especially plant protein intake such as that from lupine and fiber, may have a favorable influence on blood pressure. A 2001 study showed that the intake of 66 g/day of protein and 15 g/day of fiber had a significant additive effect in lowering blood pressure (approximately 10 mmHg) [79]. In 2009, a study was conducted by Lee et al. [72] to determine the effects on blood pressure of bread made with lupine flour (equivalent to 14 g/day of protein and 13 g/day of fiber) compared to bread made with wheat flour in overweight and obese men and women. When analyzing the results obtained, they found that the group of bread made with lupine flour showed a reduction in blood pressure of approximately 3.5 mmHg compared to those who were not in the control group [70,72,84]. This beneficial effect of lupine can be explained in part by the improvement of vascular function through the proteins that constitute it. These have a high percentage of arginine in their composition, which is the physiological substrate of endothelial nitric oxide synthase, thus enhancing the vasodilatory properties of the endothelium [84,86].

A potential mechanism that is under investigation and that was tested by Boschin et al. is the ability to inhibit the ACE (angiotensin converting enzyme) of lupine seed conglutins. Boschin et al. [87] found ACE-inhibitory activity in lupine proteins, thus offering a powerful alternative to synthetic ACE inhibitors, which can cause several side effects. Furthermore, in this study, they demonstrate that β-/α-conglutin isolate was responsible for that effect [33,87]. However, further studies are needed to understand the mechanism of action by which lupine reduces blood pressure, and investing in this research area could be a valuable opportunity to improve public health, since the reduction in blood pressure observed in these and other studies could mean a 10% difference in the prevalence of hypertension, a 4% difference in the risk for coronary artery disease and a 10% difference in the risk for stroke [70].

### 6.4. Effect on Glucose and Insulin Metabolism

Like cardiovascular diseases, diabetes is one of the most common diseases worldwide, and by 2030, an increase in the number of diagnosed cases is expected to reach 400–450 million, with 90% of cases diagnosed with type 2 diabetes [88,89]. Once again, food plays a crucial role in this disease, with pulses playing a prominent role since they provide vegetable protein and dietary fiber, have a low glycemia index and are rich in bioactive compounds, which can influence glucose metabolism [33].

Type 2 diabetes is characterized by a combination of early insulin resistance and progressive loss of pancreatic β-cell function, resulting in insufficient amounts of insulin and subsequent hyperglycemia. Therefore, an agent must maintain blood glucose levels by improving peripheral insulin sensitivity, decreasing liver glucose production, increasing insulin secretion by β cells or modulating the enzymatic activity of glucose metabolism-related enzymes such as dipeptidyl peptidase 4 (DPP4) and α-glycosidase [90].

In lupine seed, the compound that is indicated as a glucose modulator is γ-conglutin. Different studies have attributed the hypoglycemic action of γ-conglutin to its insulin-mimetic properties. Gamma-conglutin has been shown to promote the translocation of GLUT-4 receptors to the cell membrane, activate intracellular kinases and adapter proteins involved in insulin signaling, and regulate the transcription of insulin-like muscle-specific genes in vitro [90].

Among the various studies that have been carried out to prove the activity of γ-conglutin in reducing hyperglycemia, the most significant effect was observed at a concentration comparable to half a dose of metformin. Lupine protein extract proved to be 10 times more potent than standard antidiabetic medication. The work by Agrawal et al. formed the basis for developing a scalable and selective extraction process for bioactive gamma-conglutin with high yield and purity of lupine as a potential antidiabetic oral health supplement [38].

Clinical studies are currently underway that should confirm this activity in humans. This is also confirmed by Garzón-de la Mora et al., who stated that *L. albus* gamma-conglutin lowered glucose in healthy subjects and patients with type 2 diabetes mellitus [38,91].

As mentioned in Section 4.3, alkaloids, namely lupanine, can potentiate the release of insulin by glucose, becoming a tool in the treatment of type II diabetes [58].

### 6.5. Effect on Bowel Function

The role of probiotics in the regulation of intestinal flora has attracted interest in the health area, and therefore, knowledge about the importance of gastrointestinal microflora balance and the role of probiotics in health has increased [92]. Not only has knowledge increased, but so have food products with probiotic ingredients. In this area, there is evidence that lupine seed fiber can act as a prebiotic ingredient and support healthy bowel function [28,29].

According to Nordic Nutrition recommendations, adequate consumption of dietary fiber reduces the risk of constipation, which in turn reduces the risk of colorectal cancer. A dietary fiber intake of 25 to 35 g per day is recommended [37,93].

Lupine kernel fiber contains about 40% oligosaccharides, namely α-galactosides [70]. These have the potential to increase the Bifidobacterium population in the colon, which in turn supports a healthy gut. Bifidobacteria prevent the growth of pathogenic microbes and the excessive growth of detrimental microflora through production of acid, which reduces fecal pH [94]. In a study performed on 18 healthy men for 4 months, it was found that those who were given a diet containing lupine kernel fiber had a reduction in the Clostridia group bacteria in the feces, while concurrently increasing the levels of *Bifidobacterium* spp. On the same basis, another study conducted with 38 healthy men provided improved bowel function, e.g., reduced transit time, beneficially reduced stool pH and gave higher levels of butyrate (a substrate for healthy colonic cell development) without altering self-reported perceptions of gut health [28,29,81].

### 6.6. Anticonvulsant Action

Several studies have been carried out to understand the evidence of lupine use in epilepsy and the truth underneath. Studies were carried out in rats, where three extracts rich in lupine alkaloids (sparteine, cytisine, lupinine) were administered, and it was noticed that these three alkaloids produced a non-specific depressant response of the central nervous system (CNS) in rats, especially at higher doses [13].

A study carried out by a team of French researchers confirmed that lupinine, sparteine and lupine seed extract have a mild sedative action on the CNS. Furthermore, all three have been shown to delay the onset of experimentally induced seizures and increase the survival time of mice during seizure [13,14,95]. In another study where female and male Wistar rats were treated with 40 mg/kg sparteine via a metal cannula for seven consecutive days, sparteine produced 100% inhibition of maximal electrical stimulation-induced seizures on day 8 compared to 65% and 55% of inhibition rates in male and female rats that were treated with 30 mg/kg carbamazepine, respectively [82]. More recently, a study in Wistar rats obtained results that indicate the anticonvulsant effect of sparteine. In this study, sparteine (13 mg/kg, i.p.) was injected into the animals 30 min before the injection of pentylenetetrazole, pilocarpine or kainic acid, and it was found that sparteine delayed the onset and decreased the severity of convulsive behaviors in these three models of status epilepticus. Moreover, sparteine decreased the mortality of the animals treated with pentylenetetrazole and pilocarpine [59,96].

In addition to these three compounds (sparteine, cytisine, lupinine) being involved in the treatment of epilepsy, there are other theories that suggest that the high content of manganese (a metal essential for normal brain functioning; its depletion could disturb the metal’s crucial role in neurotransmission) of lupines may respond particularly to the pathophysiology of chronic epilepsy, as several studies have correlated the reduced presence of manganese in patients with frequent seizures [14].

## 7. Conclusions and Future Challenges

Since ancient times, lupine has proved to be a viable “alternative crop” in sustainable agricultural systems, being able to grow in poor, acidic, sandy soils and in adverse climatic conditions, making it an economically profitable alternative [28,29].

White lupine seeds are endowed with high protein and fiber content compared to other legumes, thus enlarging the number of sources of vegetable proteins and dietary fiber used for human nutrition. The vegetable proteins market has shown itself to be scarce and with increasingly higher costs regarding animal protein; therefore, lupine can be an alternative to fill this shortage. However, lupine has a relatively thick husk around the seed which for many people is not pleasant to consume, causing a lot of waste. Since the hull is also rich in fibers and proteins and other constituents, innovation would be important for the transformation of this, for example, into flour, where constituent minerals and phytochemicals are more bioavailable and can provide a new fiber ingredient with useful added value.

Another promising approach with a potential for high market returns is the production of specialized products for niche markets for consumers with certain needs. Globally, the nutraceutical market has been growing, especially in areas focused on cardiovascular diseases and diabetes mellitus [27]. As discussed in this paper, white lupine is endowed with favorable characteristics that would allow it to be used as a suitable nutraceutical for these diseases. Perhaps because more studies are needed in this area, to date, none of the international food safety organizations allow labeling claiming the health benefits of lupine ingredients [97]. However, if sufficient evidence exists that the beneficial properties of lupine ingredients, whether flours, protein concentrates/isolates or their fibrous fractions, are confirmed through adequate, valid and detailed clinical studies, their use as a functional or nutritional ingredient could have promising financial returns [27].

With such a promising future in terms of food and health, and even though there are already safety limits for the presence of anti-nutritional compounds and methods developed for their presence and removal, such as alkaloids, special attention is needed for the phomopsins that are still poorly studied, and the limits in the case of the European Union are not regulated. Thus, for a safe inclusion of lupine in food products and nutraceuticals, innovation is also needed to stop the development of phomopsins in lupine seeds and also to devise sophisticated methods for the removal of anti-nutritional compounds or to develop GM technologies or molecular improvement techniques. Non-GM, such as CRISPR/Cas9, may provide better solutions to meet specific lupine seed quality objectives [28,29].

Finally, striking a balance between the pros and cons, the advantages seem to have a greater price tag than the existing limitations in the lupine seeds, being quite promising. It is thus necessary for investment in the industry to expand and explore the potential of lupine seeds and their by-products, and to alert the consumer to the unique properties of lupine as a sustainable, nutritious and healthy human food ingredient.

## Figures and Tables

**Table 1:** Comparison of minerals and vitamins content of lupine seeds according to different sources, namely the United States food composition database [23] and the Portuguese food composition table [22].

Components/Sources	USDA	USDA	INSA	Other Sources
	Mature seeds, raw	Cooked lupine, without salt	Cooked lupine, without salt	
Calcium, Ca	176 mg/100 g	51 mg/100 g	48 mg/100 g	2.1–4.7 g/kg [43]
Iron, Fe	4.36 mg/100 g	1.2 mg/100 g	3.4 mg/100 g	-
Magnesium, Mg	198 mg/100 g	54 mg/100 g	54 mg/100 g	1.2–2.2 g/kg [43]
Manganese, Mn	2.38 mg/100 g	0.676 mg/100 g	-	896 mg/kg [16,32,38]
Phosphorus, P	440 mg/100 g	128 mg/100 g	110 mg/100 g	4.3–7.2 g/kg [43]
Potassium, K	1010 mg/100 g	245 mg/100 g	250 mg/100 g	8.6–11.1 g/kg [43]
Selenium, Se	9.2 µg	2.6 µg	-	-
Sodium, Na	15 mg/100 g	4 mg/100 g	4 mg/100 g	0.1–0.2 g/kg [43]
Zinc, Zn	4.75 mg/100 g	1.38 mg/100 g	1.4 mg/100 g	-
Carotenoids	0 µg/100 g	0 µg/100 g	0 µg/100 g	Reported [27,28,38]
Niacin (B3)	2.19 mg/100 g	0.495 mg/100 g	0.5 mg/100 g	Consuming 100 g of lupin satisfying 30% of B3 requirement for a diet of 2000 kcal/day [4]
Riboflavin (B2)	0.22 mg/100 g	0.053 mg/100 g	0.03 mg/100 g	Consuming 100 g of lupin satisfying 20% of B2 requirement for a diet of 2000 kcal/day [4]
Thiamin (B1)	0.64 mg/100 g	0.495 mg/100 g	0.12 mg/100 g	Consuming 100 g of lupin satisfying 50% of B1 requirement for a diet of 2000 kcal/day [4]
Tocopherol (VE)	-	-	α-tocopherol 0.1 µg/100 g	γ-tocopherol was the main isomer in *L. albus* [24]
Vitamin C	4.8 mg/100 g	1.1 mg/100 g	1.1 mg/100 g	Reported in C, was only detected in *L. albus* and *L. luteus* [28]

**Table 2:** Summary of relevant animal studies and clinical trials related to potential health benefits of lupin seeds.

Health Effect	Study Aim	No. of Participants	Duration	Model	Main Results	Ref.
**Satiety and energy intake; arterial hypertension**	Determine the effects on body weight, energy and nutrient intakes, and serum and urinary analytes of a diet moderately higher in dietary protein and fiber achieved by substituting lupin.	88	16 weeks	Randomized, controlled parallel-designed trial.	Substituting 40% lupine flour gave higher levels of self-reported satiety and lower energy intake at lunch than compared to bread made with wheat flour alone, and increasing protein and fiber in bread with lupin kernel flour may be a simple dietary approach to help reduce blood pressure and cardiovascular risk.	[72]
**Satiety and energy intake**	Measure food and energy intake two hours after the consumption of three test bread meals differing in their GI, protein, fiber and moisture content but similar in total energy.	20	120 min	Randomized controlled trial.	The subjects consumed less food following the Lupin Bread than the White Bread.	[73]
**Satiety and energy intake**	Examine the effect of replacing approximately one half of the fat in a sausage patty with inulin or lupin kernel fiber on the sensory acceptability of the products, post-meal perceptions of satiety and daily energy and fat intakes of healthy men.	33	3 weeks	Randomized within-subject trial.	Incorporation of lupine seed fiber into processed foods resulted in feelings of fullness for up to 4.5 h after ingestion and approximately 15% lower energy intake.	[74]
**Cardiovascular disease prevention**	Compare the effects of lupin protein and cysteine-supplemented casein with those of casein on atherosclerotic lesion development in apoE-deficient mice.	30 male apoE-deficient mice	16 weeks	Randomized controlled trial.	Lupin protein and cysteine-supplemented casein compared with casein reduced the calcification of atherosclerotic lesions in apoE-deficient mice.	[75]
**Cardiovascular disease prevention**	Examine the effect of a diet containing a novel legume food ingredient, Australian sweet lupin kernel fiber (LKFibre), compared to a control diet without the addition of LKFibre, on serum lipids in men.	38	1 month	Randomized crossover dietary intervention study.	Addition of LKFibre to the diet provided favorable changes to some serum lipid measures in men, which, combined with its high palatability, suggest that this novel ingredient may be useful in the dietary reduction of coronary heart disease risk.	[76]
**Cardiovascular disease prevention**	Investigate for the first time whether lupin protein also influences the lipid metabolism of lactating rats and the triglyceride content of milk by influencing SREBPs and the mRNA expression of other genes such as the peroxisome proliferator-activated receptor (PPAR)-a which is involved in the fatty acid catabolism or cholesterol 7a-hydroxylase (CYP7A1), a key enzyme for the bile acid synthesis.	24 9-week-old male Sprague–Dawley rats	-	Randomized controlled trial.	Lupin protein increases milk fat content and strongly modifies triglyceride and cholesterol metabolism by influencing the transcription levels of genes involved in fatty acid oxidation and synthesis and cholesterol homeostasis.	[77]
**Cardiovascular disease prevention**	Evaluate a possible hypolipidemic effect of lupin proteins.	30 male Sprague–Dawley rats	28 days	Randomized controlled trial.	Demonstrates a marked cholesterol-lowering activity of proteins from lupinus in rats. Moreover, lupin proteins appear to affect cellular lipid homeostasis by up-regulating SREBP-2 and CYP7A1 genes.	[78]
**Arterial hypertension**	Determine whether dietary protein and fiber had additive effects on blood pressure reduction in hypertensives.	36	4 weeks	Randomized controlled trial.	Suggested that adequate intake of protein and fiber, particularly with fruits and vegetables as sources of soluble fiber, should be considered in recommendations of an optimal diet for reduction in cardiovascular risk in subjects with normal renal function.	[79]
**Glucose and insulin metabolism**	Determine the effect of lupinus albus gamma conglutin decreases glucose in healthy subjects and type 2 diabetes mellitus patients.	31	3 days	Randomized controlled trial.	*Lupinus albus* gamma conglutins displayed a short duration hypoglycemic effects in normal volunteers and type 2 diabetic patients.	[80]
**Bowel function**	Determine the effect of LKFibre on human intestinal microbiota by quantitative fluorescent in situ hybridization (FISH) analysis.	18	4 months	Single-blind, randomized, crossover dietary intervention design.	Ingestion of LKFibre stimulated colonic bifidobacteria growth, which suggests that this dietary fiber may be considered as a prebiotic and may beneficially contribute to colon health.	[81]
**Anticonvulsant action**	Evaluated the anticonvulsant properties of sparteitn in an Epileptiform seizures Experimental Models (MECES).	100 male and female Wistar rats	8 days	Randomized controlled trial.	Sparteine produced 100% inhibition of maximal electrical stimulation-induced seizures on day 8 compared to 65% and 55% of inhibition rates in male and female rats, respectively, that were treated with 30 mg/kg carbamazepine.	[82]
